# *Lavandula angustifolia* Mill. Oil and Its Active Constituent Linalyl Acetate Alleviate Pain and Urinary Residual Sense after Colorectal Cancer Surgery: A Randomised Controlled Trial

**DOI:** 10.1155/2017/3954181

**Published:** 2017-01-05

**Authors:** So Hyun Yu, Geun Hee Seol

**Affiliations:** Department of Basic Nursing Science, School of Nursing, Korea University, Seoul 02841, Republic of Korea

## Abstract

Pain and urinary symptoms following colorectal cancer (CRC) surgery are frequent and carry a poor recovery. This study tested the effects of inhalation of* Lavandula angustifolia* Mill. (lavender) oil or linalyl acetate on pain relief and lower urinary tract symptoms (LUTS) following the removal of indwelling urinary catheters from patients after CRC surgery. This randomised control study recruited 66 subjects with indwelling urinary catheters after undergoing CRC surgery who later underwent catheter removal. Patients inhaled 1% lavender, 1% linalyl acetate, or vehicle (control group) for 20 minutes. Systolic and diastolic blood pressure (BP), heart rate, LUTS, and visual analog scales of pain magnitude and quality of life (QoL) regarding urinary symptoms were measured before and after inhalation. Systolic BP, diastolic BP, heart rate, LUTS, and QoL satisfaction with urinary symptoms were similar in the three groups. Significant differences in pain magnitude and urinary residual sense of indwelling catheters were observed among the three groups, with inhalation of linalyl acetate being significantly more effective than inhalation of lavender or vehicle. Inhalation of linalyl acetate is an effective nursing intervention to relieve pain and urinary residual sense of indwelling urinary catheters following their removal from patients who underwent CRC surgery.

## 1. Introduction

The incidence of colorectal cancer (CRC) in Korea has increased markedly due to a transition to Western dietary patterns [1]. At present, CRC is the third most common cancer in both men and women, with 27,618 patients newly diagnosed with CRC in 2013, accounting for 12.3% of the 225,343 patients newly diagnosed with cancer during that year [2]. Surgical resection is the treatment of choice for CRC, but it entails various side effects and complications. The most frequent postoperative complications include micturition disorders, ileus, changes in bowel habit, postoperative pain, enterostomy related problems, surgical site infection and bleeding, anastomotic leakage, and pulmonary complications, all of which can lead to physical pain and distress [3]. Post-CRC pain is both sharp and acute, adding to overall malaise and fatigue. Micturition disorders resulting from CRC surgery have been attributed to injuries to the iliohypogastric and/or pelvic splanchnic nervous systems [4]. The intrapelvic autonomic nervous system involved in the neural control of micturition includes the pelvic splanchnic and iliohypogastric nerves and the pelvic nervous plexus. The hypogastric nerves, which arise from the preaortic sympathetic plexus, divide to the left and right within the pelvis, forming the pelvic autonomic nerve plexus upon meeting the parasympathetic sacral splanchnic nerves that originate from the second, third, and fourth sacral foramina along the pelvic wall [5]. Therefore, any injury to the pelvic splanchnic or iliohypogastric nervous system causes a dysfunction in micturition. Injury to the parasympathetic nervous circuitry causes the sensory function of the bladder to deteriorate, increases voiding difficulty, and weakens detrusor contraction, whereas injury to the sympathetic nervous circuitry causes urinary urgency, tonic urinary incontinence, and increased urinary frequency [6]. The main neurogenic causes of micturition disorders resulting from CRC surgery may be intraoperative injuries to the ureter or pelvic autonomic nervous system. Moreover, it has been estimated that 10~15% of patients who underwent CRC surgery experience micturition disorders [7].

Efficient and immediate pain control following CRC surgery can facilitate earlier hospital discharge by reducing the length of hospital stay, thereby minimizing complications in the respiratory system, such as atelectasis and pneumonia, as well as gastrointestinal complications, including intestinal obstruction and abdominal inflation. Typical types of pain relief management after CRC surgery include patient-controlled anesthesia (PCA), narcotic (opioid) analgesics, nonsteroidal anti-inflammatory drugs, and epidural anesthesia [3]. Despite pharmacotherapy, however, many patients complain of severe pain in the area of surgery. Acute micturition disorders after CRC surgery impede return to the activities of daily life. Moreover, lower urinary tract symptoms arising from voiding difficulty degrade patient quality of life (QoL) and aggravate patient discomfort until the recovery of urinary function. To prevent this, CRC surgery, consisting of total mesorectal excision, is designed to preserve pelvic autonomic function; indeed, it was found that such autonomic nerve-preserving total mesorectal excision prevents local recurrence as well as preserving urinary functions [4].

Aromatherapy, defined as inhalation of herb oil essences, is a type of complementary and alternative therapy. Analyses of hexane extracts of* Lavandula angustifolia* Mill. (lavender) oil have shown that lavender essential oil has a diuretic effect [8]. Although lavender essential oil was less effective than benzodiazepines in mouse models of anxiety, lavender oil had an antianxiety effect [9]. Inhaled lavender oil has been shown to have relaxant effects on tracheal and ileal smooth muscles of guinea pigs and urinary bladder muscles of white mice, with their anticonvulsant activities involving cyclic AMP-dependent pathways [10]. Furthermore, an urodynamic investigation targeting female patients with urinary incontinence found that inhalation of lavender scent did not mitigate stresses induced by diuretic activity [11]. Studies with clary sage and lavender oils, the main constituent of which was linalyl acetate, showed that inhalation of clary sage oil provided pain relief to patients with periodontitis and efficiently controlled systolic blood pressure and heart rate [12]. Moreover, inhalation of clary sage oil had an antidepressive effect in mouse models of depressive behavior, via a mechanism by which clary sage oil controls dopamine activity [13].

Although lavender oil and its core constituent linalyl acetate relieve pain and anxiety reduce blood pressure and heart rate and have diuretic activity, no study to date has assessed the effect of aromatherapy with lavender and linalyl acetate on the degree of pain relief and urination satisfaction after the removal of indwelling urinary catheters. This study therefore analyzed the effects of lavender and linalyl acetate on the relief of pain in the surgery area and the level of urination satisfaction. These results may indicate an effective nursing intervention for patients following the removal of indwelling urinary catheters.

## 2. Methods

### 2.1. Study Design and Sample Size

This randomised pre- and posttest controlled trial assessed the effects of inhalation of 1% (v/v) lavender and 1% (v/v) linalyl acetate in almond oil on pain relief and lower urinary tract symptoms in patients who have undergone removal of indwelling urinary catheters after CRC surgery. Using a G-power program, it was calculated that the minimum number of subjects necessary for intergroup comparisons to achieve a significance level of 0.05, an effect size of 0.40, and test power of 0.80 was >21 subjects per group. Twenty-two (22) subjects were assigned to each group and randomised using table of random numbers to inhalation of 1% lavender oil, 1% linalyl acetate, or almond oil (used as solvent, control group).

### 2.2. Participants

The study protocol was approved by the institutional bioethics committee of Korea University Anam Hospital (ED15045). All subjects were informed about the objectives and procedures of this study, and all provided written informed consent. All patients had been diagnosed with and underwent robotic or laparoscopic surgery for CRC at Korea University Medical Center and were given postoperative fentanyl as a painkiller and ketorolac tromethamine as an anti-inflammatory agent, with all attaining a specific stable drug dosage at the time of testing. None of the included patients had any complications, inflammatory diseases, loss of consciousness, communication disorders, or disorientation after surgery. None had olfactory impairments or allergy to any of the essential oils; and none had been treated with drugs, hormones, or aromatherapy for a psychiatric disorder.

### 2.3. Procedures

Prior to testing, the selected subjects were informed about the study objectives and asked to fill out a questionnaire regarding general characteristics, VAS pain, and urination. After a 10-minute rest, blood pressure and heart rate were measured in the supine position; all measurements were taken by a single trained researcher to minimize the effects of emotional stimuli on cardiovascular responses. All subjects were not informed about the types, concentrations, and efficacy of aroma oils.

Following the removal of the indwelling urinary catheter implanted after CRC surgery, curtains were drawn to exclude the effects of experimental cues. After completing the above survey, the subjects were allowed to rest for 10 minutes and placed in the supine position. In taking three deep breaths through a 4 × 2 cm sized gauze suspended in above the philtrum of the subject, 1 ml 1% lavender oil, 1% LA, or almond oil was dropped onto the gauze, and the subject were allowed to inhale the aroma for about 20 minutes. To ensure the objectivity of this study, the researcher was not involved in formulating the inhaled oils, and testing was performed in a double blind fashion.

After each test, subjects were asked to complete a posttest survey, using the same method as that employed for the pretest survey. Subjects were allowed to rest for about 10 minutes, and blood pressure and heart rate were measured in the supine position, as above.

The main components of the lavender oil (Aromarant Co., Ltd., Rottingen, Germany), as determined by gas chromatography, were [11] linalyl acetate (38.5%), linalool (33.3%), caryophyllene (3.9%), myrcene (3.9%), trans-ocimene (2.4%), lavandulyl acetate (2.2%), and terpinen-4-ol (2.1%).

### 2.4. Pain Scale

Pain was measured by a visual analog scale (VAS) score [14]. This tool is the most common pain scale for quantification of endometriosis related pain, as it allows each respondent to directly specify his/her level of agreement using a horizontal line, usually 10 cm in length, with scores of 0 and 10 indicating no pain and the worst pain imaginable, respectively.

### 2.5. Blood Pressure and Heart Rate

Blood pressure (BP) and heart rate (HR) are parameters revealing autonomic nervous system reactions in a state of pain and were used to measure physiological reactions. BP was measured after a rest of 10 minutes or longer using an electronic manometer (3BMI-3, Microlife, Switzerland), placed on the brachial artery with the subject comfortably seated in a supine position before and after essential oil inhalation. HR was measured in the radial artery for 1 minute before and after essential oil inhalation.

### 2.6. Questionnaire regarding Lower Urinary Tract Function

Micturition function was assessed using a self-administered questionnaire that measures lower urinary tract symptoms (LUTS) based on seven items (urinary residual sense, urinary frequency, urinary intermittency, urinary urgency, urinary weak stream and urinary hesitancy, and nocturia). Each response was scored on a 5-point scale, assessing the frequency of events during the past five urination episodes, with scores of 0–5 points indicating never, 1 in 5 times, 1 in 3 times, 1 in 2 times, 2 in 3 times, and always, respectively. Scores of each of the seven items were summed to yield the total symptom score. Current urinary satisfaction as a QoL measure was scored on a 6-point scale, ranging from very happy (0 point) to terrible (6 points).

### 2.7. Statistical Analysis

Data were analyzed using SPSS 20.0 software. To preliminarily determine homogeneity across the three patient groups, categorical variables were assessed using Chi-squared or Fisher's exact tests. Continuous variables were analyzed using Kolmogorov-Smirnov tests to determine regularity, with normally distributed variables analyzed by ANOVA (analysis of variance) and nonnormally distributed variables analyzed by Kruskal-Wallis tests. Differences in dependent variables across the three groups before and after testing were analyzed by Kruskal-Wallis tests, whereas within group differences in dependent variables from before to after testing were analyzed by Wilcoxon's rank sum tests.

## 3. Results

### 3.1. General Characteristics of Subjects and Verification of Homogeneity

The mean age of the 66 subjects who participated in this study was 60.9 years. Of these subjects, 42 (63.6%) were male and 29 (43.9%) were smokers, with a mean smoking period of 11.5 years and a mean 7.2 cigarettes per day. Of these 66 subjects, 36 (54.5%) exercised every day, 33 (50%) consumed alcohol, 23 (34.8%) took antihypertensive drugs, 11 (16.7%) took hypoglycemic agents, 66 (100%) consumed at least one cup of coffee per day, 23 (34.6%) underwent robotic surgery, and 21 (31.8%) had metastatic disease. None of these factors differed significantly among the three groups (Table 1).

Prior to essential oil inhalation, the mean VAS pain score after removing indwelling urinary catheters was 4.25, mean systolic BP was 126.69 mmHg, mean diastolic BP was 73.87 mmHg, mean HR was 74.54 beats/min, mean total LUTS score was 5.44 points, and mean QoL score was 2.65 points. None of these scores differed significantly among the three groups of subjects (Table 2).

### 3.2. Effects on Pain

**Figure 1:**
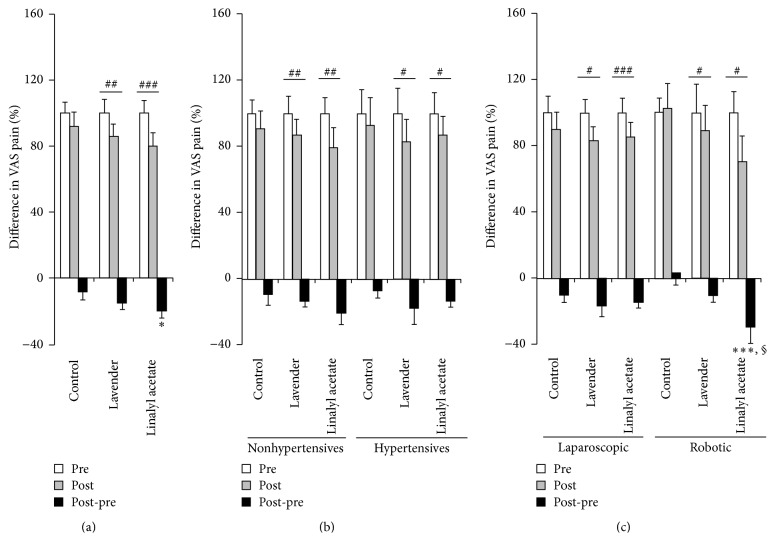
Effects of lavender or linalyl acetate inhalation on VAS pain scores in (a) all patients, (b) patients who were not treated with antihypertensive drugs (nonhypertensives) and were treated with antihypertensive drugs (hypertensives), and (c) patients who underwent laparoscopic and robotic operations. Results are presented as mean ± standard error of the mean, with differences compared by the Kruskal-Wallis test or Wilcoxon's rank sum test. ^*∗*^*P* < 0.05 versus control, ^*∗∗∗*^*P* < 0.001 versus control undergoing robotic surgery, ^§^*P* < 0.05 versus lavender undergoing robotic surgery, ^#^*P* < 0.05, ^##^*P* < 0.01, and ^###^*P* < 0.001. VAS: visual analog scale.

Pain magnitude score after essential oil inhalation differed significantly in the linalyl acetate and control groups (22 in each group, *P* = 0.035, Figure 1(a)). Relative to pretreatment levels, pain magnitude pain magnitude scores after the intervention were reduced 8.22 ± 4.77% (0.37 ± 0.21 points) in the control group, 14.39 ± 4.14% (0.56 ± 0.16 points) in the lavender group, and 19.65 ± 4.44% (0.86 ± 0.19 points) in the linalyl acetate group, with the differences between the lavender (*P* = 0.002) and linalyl acetate (*P* < 0.001) groups being significantly more reduced than in the control group (Figure 1(a)).

Pain magnitude in the three groups was not significantly affected by administration of an antihypertensive drug after the intervention (Figure 1(b)). Pain magnitude was not significantly reduced in either control subgroup (nonhypertensives, *n* = 15; hypertensives, *n* = 7) but was reduced significantly in both the lavender (nonhypertensives, *n* = 14; hypertensives, *n* = 8) and linalyl acetate subgroups (nonhypertensives, *n* = 14; hypertensives, *n* = 8).

Patients in the linalyl acetate (*n* = 7, *P* = 0.001) and lavender (*n* = 9, *P* = 0.027) groups who underwent robotic surgery showed significantly greater reductions in pain score compared with control patients who underwent robotic surgery (Figure 1(c)). Pain magnitude after the intervention increased in control patients who underwent robotic surgery (*n* = 7) but decreased in control patients who underwent laparoscopic surgery (*n* = 15). Pain magnitude in both the lavender and linalyl acetate groups decreased after both robotic (9 in the lavender group; 7 in the linalyl acetate group) and laparoscopic surgery (13 in the lavender group; 15 in the linalyl acetate group).

These findings indicate that inhalation of lavender and linalyl acetate relieved postoperative pain in patients who underwent CRC surgery. Lavender was effective in subjects who did not receive antihypertensive drugs, whereas linalyl acetate was effective in patients who did and did not receive antihypertensive drugs. Furthermore, pain relief was greater in linalyl acetate than in lavender and control patients who underwent robotic surgery.

### 3.3. Effects on BP and HR

Aroma inhalation reduced systolic BP, diastolic BP, and HR slightly, but similarly, in the three groups, with no statistically significant differences across the three groups (data unshown).

### 3.4. Effects on Urinary Symptoms and Satisfaction with Urination

Inhalation altered symptom scores slightly, but not significantly, in each of the three groups. Comparisons among the three groups showed that inhalation increased mean LUTS score by 0.63 ± 6.39 in the control group but decreased mean LUTS score by 2.83 ± 4.69 in the lavender group and 0.48 ± 7.15 in the linalyl acetate group, with no significant difference among these three groups (*P* = 0.234; Table 3). Subanalysis showed that urinary residual sense decreased more in the lavender (1.28 ± 2.08; *P* = 0.004) and linalyl acetate (0.81 ± 1.69; *P* = 0.046) groups than in the control (0.63 ± 1.67) group but that the postintervention scores were similar in the three groups (*P* = 0.540). Change in satisfaction with urination from before to after inhalation was similar in the three groups (*P* = 0.488).

## 4. Discussion

This study assessed the effects of inhalation of linalyl acetate and lavender oil on pain relief and urinary symptoms following the removal of indwelling urinary catheters from patients who underwent CRC surgery. Inhalation of linalyl acetate resulted in a significantly greater reduction in pain magnitude compared with inhalation of almond oil. Moreover, pain magnitude was significantly lower after than before inhalation of lavender and linalyl acetate.

Although antihypertensive drugs did not significantly affect pain magnitude in any of these groups, patients in the lavender and linalyl acetate groups who did and did not take antihypertensive agents showed significant reductions in pain magnitude. Patients in the linalyl acetate and lavender groups who underwent robotic surgery showed significantly greater reductions in pain score compared with control patients who underwent robotic surgery. Pain magnitude after the intervention increased in control patients who underwent robotic surgery but decreased in control patients who underwent laparoscopic surgery. Pain magnitude in both the lavender and linalyl acetate groups decreased after both robotic and laparoscopic surgery. Taken together, these findings indicate that linalyl acetate was more effective at reducing pain than lavender, consistent with reports showing the analgesic effect of linalyl acetate aromatherapy. For example, inhalation of lavender oil containing 35.35% linalyl acetate as its main component relieved migraine headaches [15]; inhalation of neroli oil containing 19.5% linalyl acetate [16] and inhalation of* Lavandula hybrida* Reverchon cv. Grosso oil containing 36.2% linalyl acetate [17] reduced peripheral and acentric pain in a mouse model of pain induced by acetic acid and a hot plate; and inhalation of bergamot essential oil containing 70.26% linalyl acetate reduced capsaicin-induced peripheral pain in mice [18].

The main aim of this study was to assess the effects of inhalation of lavender or linalyl acetate on pain relief and lower urinary tract symptoms. This implied a limitation in a small sample size which might explain the effects of lavender or linalyl acetate on lower urinary tract symptoms. Further studies with more patients may be necessary to adequately assess the effects of lavender or linalyl acetate. However, both systolic and diastolic BP were reduced in all three patient groups but tended to be reduced more in the lavender and linalyl acetate groups than in the control group, suggesting that inhalation of lavender essential oil has vasorelaxation effects. This finding is in agreement with results showing that inhalation of (R)-(−) linalool and lavender essential oils by healthy individuals reduced HR [19]. Both total symptom (LUTS) score and satisfaction at urination tended to improve more in the lavender and linalyl acetate groups than in the control group. Intragroup differences in LUTS scores, particularly in urinary residual sense, in the lavender and linalyl acetate groups suggested that inhalation affected diuretic activity, consistent with a prior study assessing the effects on BP of the diuretic action of lavender oil, as shown by urodynamic testing for urinary incontinence in women [11]. Additionally, the sample size of the subgroups of this research was relatively small. Further research undertaken for a longer duration of the study is needed. These findings suggest that inhalation of lavender oil or its main component linalyl acetate following the removal of an indwelling urinary catheter from patients with micturition disorders can reduce LUTS, especially urinary residual sense.

## 5. Conclusions

The results described in this study indicate that inhalation of essential oils containing linalyl acetate can relieve pain and urinary residual sense following the removal of indwelling urinary catheters from patients who have undergone CRC surgery. Future research should determine the concentration dependence of these effects, especially with regard to satisfaction with urination. Inhalation of linalyl acetate by these patients may be an effective nursing intervention to enhance comfort and control pain and micturition disorders.

## Figures and Tables

**Table 1:** General characteristics of the subjects (*N* = 66).

Characteristics	Control	Lavender	Linalyl acetate	*P* value
	*n* (%)	*n* (%)	*n* (%)	
Age (years)	60.64 (13.83)	61.18 (10.06)	61 (12.52)	0.989^*∗*^
Gender				0.822
Male	14 (63.64)	15 (68.18)	13 (59.09)	
Female	8 (36.36)	7 (31.82)	9 (40.91)	
Smoking				0.782
Yes	9 (40.91)	11 (50)	9 (40.91)	
No	13 (59.09)	11 (50)	13 (59.09)	
Smoking period (years)	10.95 (15.49)	15.14 (18.07)	8.86 (12.04)	0.570^‡^
Smoking amount (cigs/day)	7.36 (10.80)	8.18 (9.07)	6.05 (8.40)	0.730^‡^
Exercise frequency (times/wk)				0.177^†^
1-2	2 (9.09)	0 (0)	2 (9.09)	
3-4	2 (9.09)	5 (22.73)	3 (13.64)	
≥5	4 (18.18)	6 (27.27)	12 (54.55)	
Alcohol drinking				0.475^†^
Yes	10 (45.45)	14 (63.64)	9 (40.91)	
No	12 (54.55)	8 (36.36)	13 (59.09)	
Drinking amount (glasses/wk)				0.217^†^
1-2	5 (22.73)	1 (4.55)	2 (9.09)	
3-4	0 (0)	3 (13.64)	1 (4.55)	
5-6	1 (4.55)	1 (4.55)	2 (9.09)	
7–9	0 (0)	4 (18.18)	1 (4.55)	
≥10	4 (18.18)	5 (22.73)	3 (13.64)	
Caffeine intake (glasses/day)				0.248^†^
0-1	14 (63.64)	12 (54.55)	11 (50)	
2–4	7 (31.82)	9 (40.91)	11 (50)	
5-6	0 (0)	1 (4.55)	0 (0)	
≥7	1 (4.55)	0 (0)	0 (0)	
Antihypertensives				
Yes	7 (31.82)	8 (36.36)	8 (36.36)	0.935
No	15 (68.18)	14 (63.64)	14 (63.64)	
Surgery				
Robotic	7 (32.82)	9 (40.91)	7 (32.82)	0.774
Laparoscopic	15 (68.18)	13 (59.09)	15 (68.18)	
Oral antidiabetics				
Yes	4 (18.18)	4 (18.18)	3 (13.64)	>0.999
No	18 (81.82)	18 (81.82)	19 (86.36)	
Metastasis				
Yes	6 (27.27)	8 (36.36)	7 (31.82)	0.811
No	16 (72.73)	14 (63.64)	15 (68.18)	

Data reported as mean (standard deviation) or *n* (%).

Chi-square test.

^*∗*^Analysis of variance (ANOVA).

^‡^Kruskal-Wallis test.

^†^Fisher's exact test.

**Table 2:** Measured variables prior to essential oil inhalation in the three groups.

Variables	Group (*n*)	Mean (SD)	*P* value
VAS Pain	Control (22)	4.48 (1.42)	0.413
	Lavender (22)	3.92 (1.51)	
	Linalyl acetate (22)	4.37 (1.53)	

sBP (mmHg)	Control (22)	125.05 (18.40)	0.798^†^
	Lavender (22)	128.18 (10.48)	
	Linalyl acetate (22)	126.86 (16.52)	

dBP (mmHg)	Control (22)	74.50 (11.45)	0.453^†^
	Lavender (22)	75.27 (6.76)	
	Linalyl acetate (22)	71.86 (9.26)	

HR (beats/min)	Control (22)	77.73 (18.40)	0.524
	Lavender (22)	73.14 (11.67)	
	Linalyl acetate (22)	72.77 (15.88)	

Urinary residual sense score	Control (19)	0.79 (1.55)	0.907
	Lavender (18)	1.28 (2.08)	
	Linalyl acetate (21)	1.19 (1.89)	

Total LUTS score	Control (19)	3.74 (4.49)	0.148
	Lavender (18)	6.78 (5.84)	
	Linalyl acetate (21)	5.81 (5.44)	

Quality of life score	Control (19)	2.32 (0.67)	0.075
	Lavender (18)	2.83 (0.86)	
	Linalyl acetate (21)	2.81 (0.87)	

Data reported as mean (standard deviation)

VAS, visual analog scale; sBP, systolic blood pressure; dBP, diastolic blood pressure; HR, heart rate; LUTS, lower urinary tract symptoms.

Kruskal-Wallis test.

^†^ANOVA.

**Table 3:** Difference in total urinary symptoms and satisfaction with urination among the three groups.

Variables	Group (*n*)	Pretest	Posttest	*P* value	Difference	*P* value
Urinary residual sense score	Control (19)	0.79 (1.55)	0.16 (0.69)	0.135	−0.63 (1.67)	0.540
	Lavender (18)	1.28 (2.08)	0.00 (0.00)	0.004	−1.28 (2.08)	
	Linalyl acetate (21)	1.19 (1.89)	0.38 (1.12)	0.046	−0.81 (1.69)	

Total LUTS score	Control (19)	3.74 (4.49)	4.37 (4.86)	0.660	0.63 (6.39)	0.234
	Lavender (18)	6.78 (5.84)	3.94 (4.01)	0.059	−2.83 (4.69)	
	Linalyl acetate (21)	5.81 (5.44)	5.33 (7.23)	0.727	−0.48 (7.15)	

Quality of life score	Control (19)	2.32 (0.67)	2.32 (0.75)	>0.999	0.00 (0.82)	0.488
	Lavender (18)	2.83 (0.86)	2.50 (0.79)	0.111	−0.33 (0.77)	
	Linalyl acetate (21)	2.81 (0.87)	2.57 (0.87)	0.216	−0.24 (1.00)	

Data reported as mean (standard deviation).

LUTS: lower urinary tract symptoms.

Kruskal-Wallis test.
